# Mapping of the trans population in the Baixada Santista region, Brazil, 2023: a descriptive study

**DOI:** 10.1590/S2237-96222024v33e2024409.especial.en

**Published:** 2025-01-10

**Authors:** Barbara Iansã de Lima Barroso, Katia Cristina Bassichetto, Denise Leite Vieira, Ísis Gois, Julliana Luiz Rodrigues, Naila Janilde Seabra Santos, Paola Alves de Souza, Angela Tayra, Aline Kumow, Karin Di Monteiro Moreira, Fe Maidel, Maria Amélia de Sousa Mascena Veras, Carla Regina Mota Alonso Dièguez, Alícia Kruger, Maria Clara Gianna Garcia Ribeiro, Rosa de Alencar Souza, Carla Gianna Luppi

**Affiliations:** 1 Universidade Federal de São Paulo, Departamento de Medicina Preventiva, São Paulo, SP, Brazil; 2 Faculdade de Ciências Médicas da Santa Casa de São Paulo, São Paulo, SP, Brazil; 3 Universidade Federal de São Paulo, Núcleo de Estudos, Pesquisa, Extensão e Assistência à Pessoa Trans Professor Roberto Farina, São Paulo, SP, Brazil; 4 Secretaria de Estado da Saúde de São Paulo, Centro de Referência e Treinamento DST/Aids, São Paulo, SP, Brazil; 5 Universidade de São Paulo, Faculdade de Saúde Pública, São Paulo, SP, Brazil; 6 Universidade de São Paulo, Instituto de Psicologia, São Paulo, SP, Brazil; 7 Núcleo de Ensino e Pesquisa do Centro de Convivência É de Lei, São Paulo, SP, Brazil; 8 Coordenação de Políticas para LGBT do Município de São Paulo, São Paulo, SP, Brazil; 9 Centro de Estudos de Cultura Contemporânea, São Paulo, SP, Brazil; 10 0Ministério da Saúde. Secretaria de Vigilância em Saúde e Ambiente. Brasília, DF, Brazil

**Keywords:** Personas transgénero, Servicios de Salud para Personas Transgénero, Investigación Demográfica y de Salud, Acceso a Servicios de Salud, Poblaciones Minoritarias, Transgender People, Health Services for Transgender People, Demographic and Health Surveys, Access to Health Services, Minority

## Abstract

**Objective:**

To describe the socioeconomic and demographic characteristics of the trans population in the Baixada Santista region, São Paulo state.

**Methods:**

This was a descriptive study involving adult trans people, selected through convenience sampling in 2023. A quantitative questionnaire was administered and in-depth interviews were conducted, which were analyzed using thematic grouping.

**Results:**

A total of 237 people were recruited. Of these, 42.2% identified as trans women/*travestis* and 36.3% as trans men/ transmasculine; 65.4% were aged up to 29 years; 51.1% self-identified as White race/skin color; 52.7% were single; 80.5% had completed at least high school; 32.5% reported no income. Self-perception of transgender identity occurred predominantly between the ages of 10 and 19 (55.7%), with social transition beginning between the ages of 15 and 19 (41.8%). Fourteen in-depth interviews were conducted.

**Conclusion:**

Socioeconomic factors – education level, employment and income – are central to gender identity. Public actions and policies need to be developed and improved.

## Study contributions

**Main results:**

The trans population in the Baixada Santista region was predominantly young, comprised of White and Black individuals, and with an income below 1 minimum wage. Approximately half identified as trans women or *travestis*, and one-third were trans men or transmasculine individuals.

**Implications for services:**

The understanding of the observed social intersections, such as low income and socioeconomic vulnerability, highlights the importance of improving access to social assistance services for the transgender population of Baixada Santista and their families.

**Perspectives:**

Representative research involving sexual and gender diversity can contribute to developing public policies for this population, from the conceptualization phase to the improvement of existing policies.

## RESUMEN

**Objetivo:**

Describir las características socioeconómicas y demográficas de la población trans de la Baixada Santista.

**Métodos:**

Se trata de un estudio descriptivo, con personas trans adultas, seleccionadas por muestreo de conveniencia en 2023. Se aplicó un cuestionario cuantitativo y se realizaron entrevistas en profundidad, analizadas mediante agrupamiento temático.

**Resultados:**

Se reclutaron 237 personas. De estas, el 42,2% se identificaron como mujeres trans/travestis y el 36,3% como hombres trans/transmasculinos; el 65,4% tenía hasta 29 años de edad; el 51,1% se autodeclaró de raza/color de piel blanca; el 52,7% eran solteras; el 80,5% tenía al menos la educación secundaria completa; el 32,5% no tenía ingresos. La autopercepción de la identidad trans ocurrió mayoritariamente entre los 10 y los 19 años (55,7%), con el inicio de la transición social entre los 15 y los 19 años (41,8%). Se realizaron 14 entrevistas en profundidad.

**Conclusión:**

Los aspectos socioeconómicos –educación, empleo e ingresos– son centrales en la identidad de género. Se deben crear y mejorar acciones y políticas públicas.

## INTRODUCTION

In Brazil, data on “gender identity” are not included in representative surveys,^1^ nor in relevant national studies in the field of health.^2^ The first South American country to conduct such a survey was Uruguay..^3^

The lack of knowledge about population size and distribution according to gender identity limits the understanding of the social determinants and health disparities faced by the trans and non-binary population, making it difficult the development of effective public policies aimed at improving the quality of life of these population groups.^4^

This scenario has motivated studies to be conducted aiming to address this gap. An estimate carried out in 2021 indicated that 1.88% of the Brazilian adult population, geographically distributed across all macro-regions of Brazil, consists of trans and non-binary people.^5^ Another mapping of the trans population, carried out in São Paulo, capital city of São Paulo state, investigated the living conditions of 1,788 people, highlighting barriers to accessing education, healthcare services and the formal labor market.^6^

Although studies involving sexual and gender diversity are growing in the country, providing important support for the development of public policies, most still focus on topics such as sexual behavior and sexually transmitted infections (STIs). Studies that address social inequalities, vulnerabilities, health conditions and access to services for this population are scarce.^7-12^

There are several gaps in healthcare service for the trans population in Brazil, starting with the difficulty of access to healthcare services, especially primary care, due to stigma and structural transphobia.^13^ Furthermore, in general, existing specialized care services are unable to meet all the health demands of this population, which extend beyond body change resources.

Given this context, the objective of this work was to describe the socioeconomic and demographic profile of the trans population of the Baixada Santista region, São Paulo state.

## METHODS

### Study design and setting

A descriptive study was conducted using data from the Mapping the Trans Population of the Baixada Santista region project, a larger research project with both a quantitative and qualitative approaches. The research was carried out in the nine municipalities of the Baixada Santista region, which includes Santos and the neighboring municipalities of Bertioga, Cubatão, Guarujá, Itanhaém, Mongaguá, Peruíbe, Praia Grande and São Vicente.

During the development of the project, the involvement of individuals who shared concerns about the need for the implementation of public policies aimed at the trans population stood out. A research group was formed, including representatives from the social movement, based on previous experiences and investigations, composed of representatives from various sectors, such as universities, the Brazilian National Health System (*Sistema Único de Saúde* - SUS), human rights (municipal and state), research centers and civil society organizations.

The research followed three stages: formative, quantitative and qualitative.

The formative stage contributed to the preparation of the fieldwork: (1) key informants were identified; (2) training was provided to interviewers; and (3) partnerships were established with social movements, healthcare services and social assistance programs at the state and municipal levels, securing agreements for the use of physical spaces to conduct the interviews.

### Participants

Eligible participants were self-declared trans, *travestis*, or non-binary people, aged 18 years or older, residing, studying, or working in the Baixada Santista region, between July and November 2023. 

Participants were selected through convenience sampling via an electronic pre-registration form, adapted from the one used by the Municipal Secretariat for Human Rights and Citizenship of São Paulo. The link to the pre -registration was disseminated through social media, such as Instagram and WhatsApp, in addition to being included in printed materials with *a* QR code directing to the link. Printed invitations for participation in the research, including the access link for registration, was also disseminated in social spaces and reference points for trans people in the Baixada Santista region.

Those who responded positively to the question *Do you agree to be contacted to participate in a survey?* were contacted to schedule the interview, either in person or virtually, depending on their preference.

Active search was also conducted in cultural, health and citizenship reference locations for the trans population in the region. These included events for assisting homeless individuals, such as those organized by the Specialized Reference Center for Homeless People in São Vicente, the Instituto ProComum in Santos, and initiatives by the civil society movement in Guarujá. In addition, the research team also attended a document rectification event for trans people, promoted by Coletivo Casa das Beaut.

### Data sources and measurement

After participants agreed to participate in the research and signed the Free and Informed Consent Form (FICF), interviews were conducted, mostly by trans people (peers), either in person or remotely, via the Zoom platform, with prior schedule. Confidentiality was ensured in locations such as primary healthcare centers, specialized care services, associations, institutes, POP centers and human rights secretariats, according to the structure available in each municipality. Data collection took place between August and December 2023, and data was entered directly into the REDCap platform during the questionnaire administration.

Participation in both the quantitative and qualitative stages occurred through the provision of compensation to cover any costs incurred.

### Sampling procedures

A value of 1.88% was applied to the total adult population of the municipalities in the Baixada Santista region, the only available national estimate of the size of trans and non-binary people, obtained from a Brazilian estimate.^10^ Based on 19,965 people, with a margin of error of 5% and a design effect of 1, a sample of 377 people was calculated, proportionally distributed among the municipalities (Table 1).

**Table 1:** Table 1 – Sample size calculated for the mapping of the trans population in the Baixada Santista region, 2023

Municipalities	Estimated n	Sampled n	Registered n (%)	Participants n (%)
Bertioga	723	14	3 (21.4)	3 (21.4)
Cubatão	1,469	28	12 (42.8)	8 (28.6)
Guarujá	3,540	67	70 (104.5)	55 (82.1)
Itanhaém	1,021	19	22 (115.9)	21 (110.5)
Mongaguá	581	11	5 (45.4)	3 (27.3)
Peruíbe	665	13	4 (30.8)	4 (30.8)
Praia Grande	3,539	37	29 (78.4)	21 (56.8)
Santos	4,477	85	85 (100)	66 (77.6)
São Vicente	3,951	75	53 (70.7)	56 (74.7)
Total	19,965	377	283 (75.1)	237 (62.9)

### Questionnaire

The questionnaire was developed by adapting instruments previously used with the trans population and validated research tools to include specific topics,^16-18^ as well as self-developed questions.

The instrument covered the following topics: socioeconomic and demographic characteristics; morbidity; food and nutrition, food insecurity; body change; sexual health and STI prevention strategies; mental health; access to health services and quality of care; use of alcohol, tobacco and other psychoactive substances; experiences of discrimination, incarceration and violence; and intersectional stigma.^19-25^

### Variables

To characterize the socioeconomic and demographic profile of the study population, the following variables were selected: age group (in years – 18 and 19, 20-24, 25-29, 30-39, 40-49, 50-59, 60 and older); race/skin color (White, Black, mixed-race, Asian, Indigenous); education level (incomplete and complete elementary school I, incomplete and complete elementary school II, incomplete and complete high school, incomplete and complete higher education, postgraduate degree); current income (yes; no); total income in the previous month, equivalent to the minimum wage at the time (0; 0.1-0.4; 0.5-0.9; 1-1.9; 2-2.9; ≥ 3); housing (rented, owned, borrowed, shelter/reception centers/communal housing, homeless); current marital status (single/without a partner, married or living together, dating, separated or divorced, widowed); gender identity (trans woman/trans man, non-binary person, *travesti*, transmasculine, other); age at trans identification, in years (≤ 10, 10-14, 15-19, 20-24, 25-29, 30-34, 35-39); age at the start of social transition (10-14, 15-19, 20-24, 25-29, 30-34, 35-39, 40-49, 50 and older); and rectification of civil registry (no; yes; ongoing process; tried but failed; did not decide).

### Data analysis

A descriptive analysis was performed, presenting the variables of interest using relative and absolute frequencies.

### Qualitative research

A semi-structured interview guide was developed to allow for deeper engagement and dialogue with the data collected from the quantitative questionnaire. The guide’s questions covered six themes: the process of identification as trans; the support network; health care; mental health and quality of life; affective and/or sexual relationships; and violence.

The interviews were conducted by two trans people from the research team, trained in psychology. In addition to their familiarity with the qualitative research and prior training, the interviewers were supervised by a qualitative supervisor.

The volunteers who participated in the qualitative stage were invited to take part in an in-depth interview, scheduled for a later date, after completing the quantitative questionnaire. Efforts were made to ensure diversity in terms of gender identity, age, and place of residence/study/work.

The interviews were conducted virtually, via the Zoom platform, at a pre-scheduled date and time. They were recorded after consent and signing of the FICF. The transcription of the material and its analysis were carried out by the qualitative supervisor. After a thorough reading of the interview transcripts, the responses were grouped by similarity across the six themes that guided the interview questions.

For this article, emphasis was placed on the intersection of gender identity with socioeconomic conditions and its impact on the participants’ education, employment, and income.

### Ethical aspects

The project was approved by the Research Ethics Committees of the Centro de Referência e Treinamento em DST/Aids e da Universidade Federal de São Paulo, under opinion No. 5,971,093, on 08/12/2022, and with a Certificate of Submission for Ethical Appraisal No**.** 64010722.8.0000.5375. The participants signed the FICF in accordance with Resolution no. 466/12 of the National Health Council.

## RESULTS

**Figure 1:**
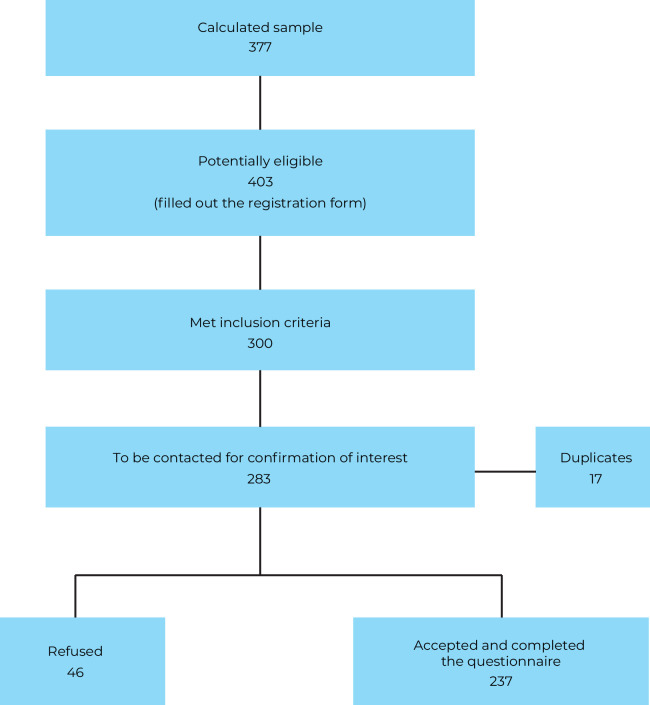
Flowchart of the capture and recruitment stages for the mapping of the trans population in the Baixada Santista region, Brazil, 2023

Of the 403 potentially eligible individuals, 300 met the inclusion criteria. After removing duplicates (n=17), 283 individuals remained to be contacted to confirm their interest in participating in the study. Of these, 237 (83.7%) agreed to participate and completed the questionnaire, representing 62.9% of the calculated sample size (377). The distribution participants in each municipality and the flowchart detailing the stages of recruitment and participant enrollment are presented in Figure 1.

Table 2 presents data on selected socioeconomic and demographic characteristics of the study participants. The majority (57.4%) were aged between 20 and 29 years; 51.1% identified as White race/skin color, while 45.1% identified as Black or mixed-race. A total of 80.5% had completed high school, of which 10.1% had completed higher education. Additionally, 32.5% of participants reported having no income; 31.2% received up to one minimum wage; 10.6% were in an unstable housing situation, living in shelters/reception centers/communal housing or in street situations; 53.0% were single or without a partner. Regarding gender identity, 42.2% identified as trans woman or *travesti*, 36.3% as trans man or transmasculine, and 20.3% as non-binary people; Furthermore, 45.6% reported recognizing their trans identity before the age of 14, and among these, 10.5% had initiated their social transition during this period.

**Table 2:** Socioeconomic and demographic characteristics of the study participants, Baixada Santista region, Brazil, 2023 (n=237)

Variables	n (%)
Age group (years)	
18-19	19 (8.0)
20-24	67 (28.3)
25-29	69 (29.1)
30-39	51 (21.5)
40-49	21 (8.9)
50-59	8 (3.4)
≥60	2 (0.8)
**Race/skin color**	
White	121 (51.1)
Mixed-race	65 (27.4)
Black	42 (17.7)
Asian	6 (2.5)
Indigenous	1 (0.4)
Does not know/did not answer	2 (0.8)
**Education level**	
Incomplete elementary school I	1 (0.4)
Complete elementary school I	3 (1.3)
Incomplete elementary school II	12 (5.1)
Complete elementary school II	8 (3.4)
Incomplete high school	22 (9.3)
Complete high school	92 (38.8)
Incomplete higher education	61 (25.7)
Complete higher education	24 (10.1)
Incomplete/complete postgraduate degree	14 (5.9)
**Current income**	
Yes	161 (67.9)
No	76 (32.1)
**Total income last month (minimum wages)**	
0	77 (32.5)
0.1-0.4	22 (9.3)
0.5-0.9	52 (21.9)
1.0-1.9	40 (16.9)
2.0-2.9	21 (8.9)
≥3	24 (10.1)
Does not know/did not answer	1 (0.4)
**Housing**	
Rented	118 (49.8)
Owned	93 (39.2)
Provided/borrowed	16 (6.8)
Shelter/reception center/communal housing	7 (3.0)
In street situations	2 (0.8)
Does not know/did not answer	1 (0.4)
**Current marital status**	
Single/without a partner	125 (52.7)
Married or living together	62 (26.2)
Dating	41 (17.3)
Separated or divorced	8 (3.4)
Widowed	1 (0.4)
**Gender identity**	
Trans woman/transsexual woman	80 (33.8)
Trans man	69 (29.1)
Non-binary	48 (20.3)
*Travesti*	20 (8.4)
Transmasculine	17 (7.2)
Other	3 (1.3)
**Age when recognized as transgender**	
<10	46 (20.3)
10-14	62 (27.3)
15-19	70 (30.8)
20-24	32 (14.1)
25-29	13 (5.7)
30-34	2 (0.9)
35-39	2 (0.9)
**Age at the start of social transition**	
10 to 14	25 (11)
15 to 19	99 (43.6)
20 to 24	67 (29.5)
25 to 29	23 (10.1)
30 to 34	6 (2.6)
35 to 39	3 (1.3)
40 to 49	2 (0.9)
50 and older	2 (0.9)
**Civil registration rectification**	
No	125 (52.7)
Yes	96 (40.5)
Ongoing process	10 (4.2)
Tried but failed	3 (1.3)
Haven’t decided	3 (1.3)

Regarding the qualitative interviews, a list of 24 potential participants, who had previously completed the quantitative questionnaire, was created. After contact, one declined to participate in the interview, and although all others initially agreed, nine did not attend at the scheduled time, even after follow-up attempts. Thus, a total of 14 in-depth interviews were conducted, with an average duration of 58 minutes each. The longest interview lasted 1 hour and 44 minutes, while the shortest lasted 32 minutes.

The participants were aged between 18 and 45 years old; three identified as trans men, three as non-binary, seven as trans women and one as a *travesti*; six people identified as mixed-race, two as Black, five as White and one chose not to disclose their race. They lived or worked in the municipalities of São Vicente, Itanhaém, Santos, Praia Grande, Guarujá and Cubatão.

The key themes related to social and economic aspects discussed during the interviews are presented in Table 3.

**Table 3:** Selected excerpts from participant narratives, related to fictitious names, gender identity, race/skin color and age, Baixada Santista, Brazil, 2023

Theme	Excerpt
Age at gender transition	*“My transition started when I was 13 years old, when my father kicked me out because of my gender identity (...) Where I went to live with other trans women, who were much older than me at that time (...)”.* (Ágata, trans woman, White, 45 years old) *“Until I was 16, I only understood that I was a very eccentric person and that I was different from everyone else (...) I watched a video (...) about non-binary genders.* *And then I watched this video when I was 17, I think. I watched this video. The was a 15-minute video, I don’t know.* *In 15 minutes I understood everything. When the video ended, I said, “Oh, this is what I am.”* (Ariel, non-binary, White, 26 years old) *“(...) it was complicated for me. Then I wanted to come out and my family accepted me as a homosexual, but they didn’t accept me as trans, they didn’t accept me dressing as a woman.* *So,* *in order to be able to be what I wanted, I had to leave my home. (...) I left home when I was 12 years old (...)”.* (Letícia, trans woman, Black, 35 years old)
Education	*“(...) during my school years I was mistreated a lot, nowadays we talk about* bullying*, but back then they mocked me, they really beat me, so it wasn’t just verbal* bullying, it was also physical attack*. However, because I couldn’t finish my studies, I dropped out in the sixth grade of* *elementary school. I plan to go back one day, who knows (...)”.* (Sofia, *travesti*, mixed-race, 43 years old) *“I am a pharmacist and biochemist, I never had any problems. At the time, in college, I had to deal with a lot of annoying things, you know, because of the bathroom use, you know, things like that.”* (Ingrid, trans woman, White, 40 years old)
Income – Challenges in employment due to gender identity	*“So, there’s this thing about not being hired, ‘like, oh,* *she’s a* travesti*’, ‘oh, no, that faggot is too flamboyant’, oh, I don’t know what* ‘ *(...)* *At my last salon job (...) I quit because of transphobia, because the person already knew my social name, but I hadn’t legally changed it yet, and she insisted on calling me by my legal name*.” (Sofia) *And I went to a job interview at a* *n employment agency (...). There was an opening, where I met all the requirements for the position, and in the interview they said... It was clear, you know? It wasn’t explicitly said, but like: oh, do you have a postgraduate degree in that? I said, no, but I’ve worked with that, I can prove it with my employment record. Oh, but you need to. There was no discrimination in any of that, it was clear, you know? That, like, they didn’t want me, you know?”* (Ingrid) *“Because I lost my job. Like, right? Things started getting really trans for me. And then I lost my job.”* (Eller, non-binary/trans person, White, 27 years old)

## DISCUSSION

This study, the first of its kind conducted in Baixada Santista, described the socioeconomic and demographic profile of the trans population residing, working or studying in the region.

The mapping of the transgender population in the Baixada Santista region employed an online recruitment methodology, which proved to be easy to organize and low-cost. The response to the invitation was positive, with a significant number of individuals completing the initial registration. However, in some municipalities in the Baixada Santista region, the number of participants was lower than expected. It is possible that the distribution of the trans population is heterogeneous in this region, and factors such as the activity of the social movement and the presence of health and social assistance networks may have influenced participation.

One in five participants identified as non-binary, a proportion higher than that found in a study by the Center for Contemporary Cultural Studies (*Centro de Estudos de Cultura Contemporânea* - CEDEC - 2021), conducted in the city of São Paulo in 2021.^6^ Differences in self-identification regarding gender may arise from the challenges of capturing this variable in research, given its fluid nature and, in some cases, the transitional stage individuals may be experiencing. In addition, generational and temporal factors may have contributed to a proportion of trans men and transmasculine individuals closer to that of trans women and *travesti^s^*.^6^ Another factor that may have contributed to this difference was the alternative method of recruitment through online registration.^24^

The proportion of people aged up to 29 years was higher in the Baixada Santista region, around two-thirds compared to the population surveyed in São Paulo (55%).^6^ The online pre-registration may have led to a greater participation of younger respondents observed in this study.

The processes of self-perception of transgender identity and the beginning of social transition occurred mainly between the ages of 10 and 19 and 15 and 19, respectively. This result corroborates data from a study conducted in 2021 in the city of São Paulo, which identified that 70% of participants had perceived their gender identity between the ages of 5 and 15.^6^

The population that identified as Black/mixed-race in the Baixada Santista region was smaller than that observed in the mapping conducted by CEDEC^6^ (42.2% *versus* 57.0%), but larger than that found in the TransOdara /São Paulo study (24.7%).^25^

More than 80% of participants had completed high school, a figure higher than the results observed in the mapping of the transgender population in the city of São Paulo (63%) and the TransOdara /São Paulo study (75^%^)^.6^.^25^ This proportion is also higher than that of the general population aged 25 or over (54.5%).^26^

About one-third of the participants reported having no income in the previous month, a figure higher than that found in the CEDEC study (6%). The proportion of people who reported an income greater than 3 minimum wages (10.1%) was double that found in the survey conducted by CEDEC (5.0%)^6^ and the TransOdara/São Paulo (4.3%).^25^

One-tenth of the participants lived in unstable housing conditions, a percentage lower than that observed in other comparative surveys (18% and 13%, respectively).^6^, These previous studies took place during the covid-19 pandemic, which may have influenced the difference in the data obtained.^6^

Issues related to schooling, employment, and housing were also present in the qualitative phase. Two trans women interviewed reported that they had left or expelled from their homes due to gender issues, at the ages of 12 and 13, respectively. In addition, there were reports of difficulty remaining in school and at work due to situations of verbal and/or physical violence. The National Association of *Travestis* and Transsexuals (*Associação Nacional de Travestis e Transexuais* - ANTRA) estimates that 13 is the average age at which trans women and *travestis* are expelled from their homes by their parents.^27^ Understanding and expressing gender identity transforms the experience of trans people, who may begin to experience social marginalization from their families,^6^ which is related to school dropout of school and difficulty in accessing the formal labor mark^et.^ 27,9

The excerpts from the qualitative phase highlighted that socioeconomic aspects – education level, employment and income – are centrally influenced by gender identity, based on participants’ perceptions. Therefore, research, actions and public policies that target education, employment and income among this population must consider variables related to gender identity, such as violence in family, school, and work environments, which impact access to and retention in the job market, as well as income.^22^, , It is also important to highlight the importance of improving access for trans people, both adolescents and adults, to services associated with social assistance policies, to prevent situations of social vulnerability and violation of rights,^29^ while understanding the impact of transphobia on the work-income relationship.^29^

The Mapping of the Trans Population of the Baixada Santista achieved its objective of providing data on the socioeconomic and demographic characteristics of the trans population of the Baixada Santista region. 

In this study, an online recruitment method was predominantly used, through filling out a registration form, whose advantages were: ease of application, dissemination and low cost; and the potential of greater reach, especially among the adult population. However, one of the disadvantages was the difficulty of reaching individuals who are unfamiliar with or lack access to the internet. The methodology used in this study proved effective in capturing the sample, although, in some municipalities, the number of people included was lower than expected, despite the research team’s exhaustive efforts in dissemination. The limitations of the study include, in addition to the disadvantages of recruitment, a non-probabilistic sample of the trans population with spontaneous participation. Therefore, the results obtained in this research may not be representative of the entire trans population.

This study assessed some socioeconomic aspects in a sample of trans people in the Baixada Santista region, characterized by a predominantly young population, with a majority identifying as White or Black, and a more balanced gender identity distribution. There was a high frequency of high school completion, but more than 60% of people had no income or earned less than 1 minimum wage. Additionally, the qualitative results revealed that social aspects related to transgender identity pose obstacles to educational level and entry into the labor market.
