# Supplementary material for: Mapping of the trans population in the Baixada Santista region, Brazil, 2023: a descriptive study
Source: Epidemiol Serv Saude. 2025 Jan 10;33(spe1):e2024409. doi: 10.1590/S2237-96222024v33e2024409.especial.en (PMC11734653; doi:10.1590/S2237-96222024v33e2024409.especial.en)
Supplement: Translation

# Translation: Mapeamento da população trans da Baixada Santista, 2023: estudo descritivo

**DOI:** 10.1590/S2237-96222024v33e2024409.especial.pt

**Authors:** Barbara Iansã de Lima Barroso, Katia Cristina Bassichetto, Denise Leite Vieira, Ísis Gois, Julliana Luiz Rodrigues, Naila Janilde Seabra Santos, Paola Alves de Souza, Angela Tayra, Aline Kumow, Karin Di Monteiro Moreira, Fe Maidel, Maria Amélia de Sousa Mascena Veras, Carla Regina Mota Alonso Dièguez, Alícia Kruger, Maria Clara Gianna Garcia Ribeiro, Rosa de Alencar Souza, Carla Gianna Luppi

**Affiliations:** 1 Universidade Federal de São Paulo, Departamento de Medicina Preventiva, São Paulo, SP, Brasil; 2 Faculdade de Ciências Médicas da Santa Casa de São Paulo, São Paulo, SP, Brasil; 3 Universidade Federal de São Paulo, Núcleo de Estudos, Pesquisa, Extensão e Assistência à Pessoa Trans Professor Roberto Farina, São Paulo, SP, Brasil; 4 Secretaria de Estado da Saúde de São Paulo, Centro de Referência e Treinamento DST/Aids, São Paulo, SP, Brasil; 5 Universidade de São Paulo, Faculdade de Saúde Pública, São Paulo, SP, Brasil; 6 Universidade de São Paulo, Instituto de Psicologia, São Paulo, SP, Brasil; 7 Núcleo de Ensino e Pesquisa do Centro de Convivência É de Lei, São Paulo, SP, Brasil; 8 Coordenação de Políticas para LGBT do Município de São Paulo, São Paulo, SP, Brasil; 9 Centro de Estudos de Cultura Contemporânea, São Paulo, SP, Brasil; 10 0Ministério da Saúde, Secretaria de Vigilância em Saúde e Ambiente. Brasília, DF, Brasil

**Keywords:** Pessoas transgênero, Serviços de Saúde para Pessoas Transgênero, Pesquisa Demográfica e de Saúde, Acesso a Serviços de Saúde, Populações Minoritárias

## Abstract

**Objetivo:**

Descrever as características socioeconômicas e demográficas da população trans da Baixada Santista.

**Métodos:**

Trata-se de estudo descritivo, com pessoas trans adultas, selecionadas por amostra de conveniência em 2023. Foi aplicado questionário quantitativo e realizadas entrevistas em profundidade, analisadas por agrupamento temático.

**Resultados:**

Foram recrutadas 237 pessoas. Dessas, 42,2% se identificaram como mulher trans/travesti e 36,3% como homem trans/transmasculino; 65,4% tinham até 29 anos de idade; 51,1% se autodeclararam de raça/cor da pele branca; 52,7% eram solteiras; 80,5% tinham, no mínimo, o ensino médio completo; 32,5% não tinham renda. A autopercepção da identidade trans ocorreu majoritariamente entre 10 e 19 anos (55,7%), com início da transição social entre 15 e 19 anos (41,8%). Foram realizadas 14 entrevistas em profundidade.

**Conclusão:**

Os aspectos socioeconômicos – escolaridade, emprego e renda – estão na centralidade da identidade de gênero. Ações e políticas públicas precisam ser criadas e aprimoradas.

## Contribuições do estudo

**Principais resultados:**

A população trans da Baixada Santista se caracterizou por ser majoritariamente jovem, branca e negra, e com renda menor do que 1 salário mínimo. Cerca de metade eram mulheres trans ou travestis, e um terço, homens trans ou transmasculinos.

**Implicações para os serviços:**

A compreensão sobre os atravessamentos sociais observados, como a baixa renda e a vulnerabilização socioeconômica, indicam a importância de melhorar o acesso aos serviços de assistência social à população trans da Baixada Santista e suas famílias.

**Perspectivas:**

Pesquisas representativas envolvendo diversidade sexual e de gênero podem contribuir para embasar políticas públicas para esta população, desde o processo de sua idealização até melhorias das políticas vigentes.

## INTRODUÇÃO 

No Brasil, o dado sobre “identidade de gênero” não consta em inquéritos representativos,^1^ tampouco em estudos nacionais relevantes na área da saúde.^2^ O país da América do Sul pioneiro nesse levantamento foi o Uruguai.^3^

O desconhecimento do tamanho e da distribuição populacional segundo identidade de gênero limita a compreensão dos determinantes sociais e das disparidades de saúde enfrentadas pela população trans e não-binária, dificultando o desenvolvimento de políticas públicas eficazes para a melhoria da qualidade de vida desses grupos populacionais.^4^

Este cenário tem motivado a realização de estudos, com o objetivo de suprir tal lacuna. Estimativa realizada em 2021 apontou que 1,88% da população adulta brasileira, distribuída geograficamente em todas as macrorregiões brasileiras, corresponde a pessoas trans e não-binárias.^5^ Outro mapeamento da população trans, realizado no município de São Paulo, investigou as condições de vida de 1.788 pessoas, evidenciando barreiras de acesso à educação, aos serviços de saúde e ao mercado de trabalho formal.^6^

Embora os estudos envolvendo diversidade sexual e de gênero estejam crescendo no país, fornecendo subsídios importantes à elaboração de políticas públicas, a maioria ainda foca temas como o comportamento sexual e infecções sexualmente transmissíveis (IST). Raros são os estudos que abordam as desigualdades e vulnerabilidades sociais, condições de saúde e acesso aos serviços dessa população.^7-12^

Há várias lacunas na assistência à saúde da população trans no Brasil, começando pela dificuldade de acesso de pessoas trans a serviços de saúde, especialmente de atenção primária, devido ao estigma e à transfobia estrutural.^13^ Além disso, de forma geral, os serviços de atenção especializada existentes não conseguem atender a todas as demandas em saúde dessa população, que vão além dos recursos de modificação corporal.

Considerando este contexto, o objetivo deste trabalho foi descrever o perfil socioeconômico e demográfico da população trans da Baixada Santista.

## MÉTODOS

### Delineamento e contexto

Foi realizado um estudo descritivo a partir de dados do projeto Mapeamento da População Trans da Baixada Santista, uma pesquisa maior com abordagem quantitativa e qualitativa. A pesquisa foi realizada nos nove municípios da Baixada Santista, que inclui Santos e os municípios vizinhos Bertioga, Cubatão, Guarujá, Itanhaém, Mongaguá, Peruíbe, Praia Grande e São Vicente. 

Durante a construção do projeto, destacou-se a participação de pessoas que compartilhavam preocupações sobre a necessidade de implementação de políticas públicas voltadas à população trans. Formou-se um grupo de pesquisa que incluía representantes do movimento social, com base em experiências e investigações anteriores, composto por representantes de diversos segmentos, como universidade, Sistema Único de Saúde (SUS), direitos humanos (municipal e estadual), centros de pesquisa e organizações da sociedade civil. 

A investigação seguiu três etapas: formativa, quantitativa e qualitativa.

A formativa contribuiu para preparar o trabalho de campo: (1) identificaram-se informantes-chaves; (2) realizou-se treinamento das pessoas entrevistadoras; e (3) firmaram-se parcerias com os movimentos sociais, serviços de saúde e assistência social, estadual e dos municípios envolvidos, com pactuação para utilização de espaços físicos para a realização das entrevistas.

### Participantes

Foram elegíveis pessoas trans, autodeclaradas como trans, travesti ou não-binária, com 18 anos ou mais, que residiam, estudavam ou trabalhavam na região da Baixada Santista, entre julho e novembro de 2023. 

As pessoas que participaram do estudo foram selecionadas por meio de amostragem de conveniência, a partir de um pré-cadastro eletrônico, adaptado de formulário utilizado pela Secretaria Municipal de Direitos Humanos e Cidadania de São Paulo. O *link* para o pré-cadastro foi divulgado por meio de mídias sociais, como Instagram e WhatsApp, além de constar em impressos com *QR-code* que direcionava ao *link*. Um convite impresso para participação na pesquisa, incluindo o *link* de acesso para o cadastro, também foi divulgado em locais de socialização e referência para as pessoas trans da Baixada Santista.

As que responderam positivamente à pergunta *Você aceita que entremos em contato para participar de uma pesquisa?* foram contatadas para agendamento da entrevista, de acordo com a modalidade escolhida, presencial ou virtual.

Foram realizadas também buscas ativas em locais de referência cultural, de saúde e de cidadania voltados à população trans da região. Entre esses, destacam-se eventos de assistência a pessoas em situação de rua, como ações do Centro de Referência Especializado para População em Situação de Rua de São Vicente, o Instituto ProComum, em Santos, e as iniciativas do movimento da sociedade civil, no Guarujá. Além disso, a equipe de pesquisa também esteve presente em um mutirão de retificação de documentos para pessoas trans, promovido pelo Coletivo Casa das *Beauty*.

### Fontes de dados e mensuração

Após a concordância em participar da pesquisa e a assinatura do Termo de Consentimento Livre e Esclarecido (TCLE), as entrevistas foram conduzidas, em sua maioria, por pessoas trans (pares), de forma presencial ou remota, via plataforma Zoom, com agendamento prévio. Garantiu-se um ambiente de confidencialidade em locais como unidades básicas de saúde, serviços de atenção especializada, associações, institutos, centros POP e secretarias de direitos humanos, conforme a estrutura disponível em cada município. A coleta de dados ocorreu entre agosto e dezembro de 2023, com os dados sendo inseridos diretamente na plataforma REDCap durante a aplicação dos questionários.

A participação nas etapas quantitativa e qualitativa ocorreu mediante fornecimento de ressarcimento para cobrir os custos envolvidos.

### Procedimentos de amostragem

Foi aplicado o valor de 1,88% sobre o total da população adulta dos municípios pertencentes à Baixada Santista, única estimativa nacional disponível da magnitude de pessoas trans e não-binárias, obtida a partir de uma estimativa brasileira.^10^ Com base em 19.965 pessoas, com margem de erro de 5% e efeito de desenho de 1, calculou-se uma amostra de 377 pessoas, proporcionalmente distribuída entre os municípios (Tabela 1).

**Tabela 1:** Tamanho da amostra calculada referente ao mapeamento da população trans da Baixada Santista, 2023

Municípios	Estimada n	Amostrada n	Cadastrada n (%)	Participantes n (%)
Bertioga	723	14	3 (21,4)	3 (21,4)
Cubatão	1.469	28	12 (42,8)	8 (28,6)
Guarujá	3.540	67	70 (104,5)	55 (82,1)
Itanhaém	1.021	19	22 (115,9)	21 (110,5)
Mongaguá	581	11	5 (45,4)	3 (27,3)
Peruíbe	665	13	4 (30,8)	4 (30,8)
Praia Grande	3.539	37	29 (78,4)	21 (56,8)
Santos	4.477	85	85 (100)	66 (77,6)
São Vicente	3.951	75	53 (70,7)	56 (74,7)
Total	19.965	377	283 (75,1)	237 (62,9)

### Questionário

Para a elaboração do questionário, foram adaptados instrumentos já utilizados com população trans e instrumentos de pesquisas já validados, para a inclusão de temas específicos,^16-18^ além de questões de elaboração própria. 

O instrumento contemplou os seguintes temas: características socioeconômicas e demográficas; morbidade; alimentação e nutrição, insegurança alimentar; modificação corporal; saúde sexual e estratégias de prevenção às IST; saúde mental; acesso a serviços de saúde e qualidade da assistência; uso de álcool, tabaco e outras substâncias psicoativas; experiências de discriminação, encarceramento e violência; e estigma interseccional.^19-25^

### Variáveis 

Para a caracterização socioeconômica e demográfica da população do estudo, foram selecionadas as seguintes variáveis: faixa etária (em anos – 18 e 19, 20-24, 25-29, 30-39, 40-49, 50-59, 60 e mais); raça/cor de pele (branca, preta, parda, amarela, indígena); escolaridade (ensino fundamental I incompleto e completo, ensino fundamental II incompleto e completo, ensino médio incompleto e completo, ensino superior incompleto e completo, pós-graduação); renda atual (sim; não); renda total no último mês, equivalente ao salário mínimo da época (0; 0,1-0,4; 0,5-0,9; 1-1,9; 2-2,9; ≥ a 3); moradia (alugada, própria, cedida/emprestada, albergue/centro de acolhida/república, na rua); situação conjugal atual (solteira/sem parceria, casada ou morando junto, namorando, separada ou divorciada, viúva); identidade de gênero (mulher trans/mulher transexual, homem trans, não-binária, travesti, transmasculino, outra); idade em que se percebeu trans, em anos (≤ 10, 10-14, 15-19, 20-24, 25-29, 30-34, 35-39); idade do início da transição social (10-14, 15-19, 20-24, 25-29, 30-34, 35-39, 40-49, 50 e mais); e retificação de registro civil (não; sim; processo em andamento; tentou, mas não conseguiu; não decidiu). 

### Análise dos dados

Foi realizada análise descritiva, com apresentação das variáveis de interesse por meio de frequências relativas e absolutas.

### Pesquisa qualitativa

Para sua execução, foi elaborado um roteiro de entrevista semiestruturado para possibilitar aprofundamento e interlocução com os dados levantados no questionário quantitativo. As perguntas do roteiro contemplaram seis temas: o processo de identificação como pessoas trans; a rede de apoio; os cuidados à saúde; a saúde mental e qualidade de vida; relacionamentos afetivos e/ou sexuais; e violência. 

As entrevistas foram conduzidas por duas pessoas trans da equipe de pesquisa, com formação em psicologia. Além de proximidade com o campo qualitativo e de treinamento prévio, as pessoas entrevistadoras foram acompanhadas pela supervisora qualitativa. 

As pessoas voluntárias que participaram desta etapa qualitativa foram convidadas a participar de uma entrevista em profundidade, agendada para outro momento, depois de terem respondido ao questionário quantitativo. Para a realização do convite, buscou-se atender à maior diversidade quanto à identidade de gênero, idade e local de residência/estudo/trabalho. 

As entrevistas foram realizadas de modo virtual, por meio da plataforma Zoom, em dia e horário previamente agendados. Foram gravadas após o consentimento e assinatura do TCLE. A transcrição do material e sua análise foi efetivada pela supervisora qualitativa. Após leitura minuciosa da transcrição das entrevistas, buscou-se identificar e agrupar por semelhança e aproximação nos seis temas que nortearam as perguntas da entrevista. 

Para este artigo, destacou-se o atravessamento da categoria identidade de gênero nas condições socioeconômicas e seu impacto na escolaridade, trabalho e renda das pessoas participantes.

### Aspectos éticos

O projeto foi aprovado nos Comitês de Ética em Pesquisa do Centro de Referência e Treinamento em DST/Aids e da Universidade Federal de São Paulo, sob o parecer nº 5.971.093, em 8/12/2022, e com Certificado de Apresentação de Apreciação Ética n**º** 64010722.8.0000.5375. As pessoas participantes assinaram o TCLE conforme a resolução nº 466/12 do Conselho Nacional de Saúde.

## RESULTADOS 

Das 403 pessoas potencialmente elegíveis, 300 atenderam aos critérios de inclusão. Ao se retirarem as duplicidades (n=17), restaram 283 a serem contactadas, para confirmação de interesse em participar do estudo. Dessas, 237 (83,7%) aceitaram participar e responderam ao questionário, representando 62,9% da amostra calculada (377). A distribuição das pessoas que participaram do estudo em cada município e o fluxograma das etapas de captação e recrutamento são apresentados na Figura 1.

**Figura 1:** Fluxograma das etapas de captação e recrutamento do mapeamento da população trans da Baixada Santista, 2023

A Tabela 2 apresenta os dados relativos às características socioeconômicas e demográficas selecionadas dos participantes do estudo. A maioria (57,4%) tinha entre 20 e 29 anos de idade; 51,1% se identificaram como sendo de raça/cor de pele branca e 45,1% como pretas/pardas; 80,5% completaram o ensino médio, entre as quais 10,1% tinham superior completo. Declararam não ter renda 32,5% das pessoas participantes; 31,2% recebiam até 1 salário mínimo; 10,6% se encontravam em situação de moradia instável, vivendo em albergues/centros de acolhida/república ou na rua; 53,0% eram pessoas solteiras ou sem parceria(s). Quanto à identidade de gênero, 42,2% se declararam como mulher trans ou travesti, 36,3% como homem trans ou transmasculino, e 20,3% como pessoa não-binária; 45,6% informaram ter se percebido como pessoa trans antes dos 14 anos, e dessas, 10,5% iniciaram sua transição social ainda nessa fase. 

**Tabela 2:** Características socioeconômicas e demográficas das pessoas participantes do estudo, Baixada Santista, 2023 (n=237)

**Variáveis**	**n (%)**
Faixa etária (anos)	
18-19	19 (8,0)
20-24	67 (28,3)
25-29	69 (29,1)
30-39	51 (21,5)
40-49	21 (8,9)
50-59	8 (3,4)
≥60	2 (0,8)
**Raça/cor da pele**	
Branca	121 (51,1)
Parda	65 (27,4)
Preta	42 (17,7)
Amarela	6 (2,5)
Indígena	1 (0,4)
Não sabe/não respondeu	2 (0,8)
**Escolaridade**	
Ensino fundamental I incompleto	1 (0,4)
Ensino fundamental I completo	3 (1,3)
Ensino fundamental II incompleto	12 (5,1)
Ensino fundamental II completo	8 (3,4)
Ensino médio incompleto	22 (9,3)
Ensino médio completo	92 (38,8)
Ensino superior incompleto	61 (25,7)
Ensino superior completo	24 (10,1)
Pós-graduação incompleta/completa	14 (5,9)
**Renda atual**	
Sim	161 (67,9)
Não	76 (32,1)
**Renda total do último mês (salários mínimos)**	
0	77 (32,5)
0,1-0,4	22 (9,3)
0,5-0,9	52 (21,9)
1,0-1,9	40 (16,9)
2,0-2,9	21 (8,9)
≥3	24 (10,1)
Não sabe/não respondeu	1 (0,4)
**Moradia**	
Alugada	118 (49,8)
Própria	93 (39,2)
Cedida/emprestada	16 (6,8)
Albergue/centro de acolhida/república	7 (3,0)
Na rua	2 (0,8)
Não sabe/não respondeu	1 (0,4)
**Situação conjugal atual**	
Solteira/sem parceria	125 (52,7)
Casada ou morando junto	62 (26,2)
Namorando	41 (17,3)
Separada ou divorciada	8 (3,4)
Viúva	1 (0,4)
**Identidade de gênero**	
Mulher trans/mulher transexual	80 (33,8)
Homem trans	69 (29,1)
Não-binária	48 (20,3)
Travesti	20 (8,4)
Transmasculino	17 (7,2)
Outra	3 (1,3)
**Idade em que se percebeu como pessoa trans**	
<10	46 (20,3)
10-14	62 (27,3)
15-19	70 (30,8)
20-24	32 (14,1)
25-29	13 (5,7)
30-34	2 (0,9)
35-39	2 (0,9)
**Idade de início da transição social**	
10 a 14	25 (11)
15 a 19	99 (43,6)
20 a 24	67 (29,5)
25 a 29	23 (10,1)
30 a 34	6 (2,6)
35 a 39	3 (1,3)
40 a 49	2 (0,9)
50 e mais	2 (0,9)
**Retificação do registro civil**	
Não	125 (52,7)
Sim	96 (40,5)
Processo em andamento	10 (4,2)
Tentou, mas não conseguiu	3 (1,3)
Não decidiu	3 (1,3)

Com relação às entrevistas qualitativas, criou-se uma listagem com 24 potenciais participantes que já haviam respondido ao questionário quantitativo. Após contato, uma recusou-se a participar da entrevista; todas as demais aceitaram, porém nove não compareceram na data e horário agendados, mesmo após outras tentativas. Assim, foi realizado um total de 14 entrevistas em profundidade, com duração média de 58 minutos cada. A mais extensa levou 1 hora e 44 minutos, e a mais breve, 32 minutos.

As pessoas participantes tinham entre 18 e 45 anos; três se identificaram como homens trans, três não-binárias, sete mulheres trans e uma travesti; seis pessoas se autorreferiram como pardas, duas como pretas, cinco como brancas e uma preferiu não se identificar. Residiam ou trabalhavam em São Vicente, Itanhaém, Santos, Praia Grande, Guarujá e Cubatão. 

Os principais temas relacionados a aspectos sociais e econômicos trazidos durante as entrevistas estão apresentados no Quadro 1. 

**Quadro 1:** Excertos selecionados de narrativas das pessoas entrevistadas, com referência aos nomes fictícios, identidade de gênero, raça/cor e idade, Baixada Santista, 2023

Tema	Excerto
Idade da transição de gênero	*“A minha transição começou aos 13 anos de idade, que meu pai me pôs para fora devido à minha identidade de gênero (...) Onde eu fui morar com outras mulheres trans, já bem mais velhas do que eu naquela época (...)”.* (Ágata, mulher trans, branca, 45 anos) *“Até os meus 16 anos, eu só entendia que eu era uma pessoa muito excêntrica e que eu era diferente de todo mundo (...) eu assisti um vídeo (...) sobre gêneros não-binários. E daí eu assisti esse vídeo acho que já com 17 (anos). Eu assisti esse vídeo. O vídeo tinha, sei lá, 15 minutos. Em 15 minutos eu entendi tudo. Quando o vídeo acabou eu falei ah, é isso que eu sou.”* (Ariel, pessoa não-binária, branca, 26 anos) *“(...) foi complicado pra mim. Aí eu quis se assumir e minha família me aceitava eu ser homossexual, mas não aceitava eu ser trans, não aceitava eu vestir roupa de mulher. Então, pra mim poder ser o que eu queria, eu tive que sair da minha casa. (... ) Eu saí de casa com 12 anos de idade (...)”.* (Letícia, mulher trans, negra, 35 anos)
Escolaridade	*“(...) no período escolar eu fui muito maltratada, hoje em dia se fala* bullying*, mas antigamente tiravam sarro mesmo, batiam mesmo, então não era só um* bullying *falado, era um* bullying *de ataque também. No entanto, porque eu não consegui terminar meus estudos, eu parei na sexta série do ensino fundamental. Pretendo voltar um dia, quem sabe (...)”.* (Sofia, travesti, parda, 43 anos) *“Eu sou farmacêutica e bioquímica, nunca tive problema. Na faculdade, na época, eu passei bastante coisinhas chatas, né, por conta de banheiro, enfim, umas coisas assim.”* (Ingrid, mulher trans, branca, 40 anos)
Renda – dificuldade no acesso e permanência no emprego em função da identidade de gênero	*“Então, tem essa coisa de não ser contratada, ‘ah, ela é travesti’, ‘ah, não, essa bicha é muito pintosa” ah, eu não sei o quê*’ *(...) No meu último salão (...) eu saí por conta de uma transfobia, porque a pessoa já sabia o meu nome social, só que eu ainda não tinha retificação, e ela fazia a questão de me chamar pelo nome ali do documento”*. (Sofia) *E eu fui a uma entrevista, numa agência de emprego (...). Tinha uma vaga, onde eu tinha todos os requisitos da vaga, e na entrevista foi colocar... Era nítida, entendeu? Não foi claramente dito, mas, assim: ah, você tem pós-graduação nisso? Falei, não, mas eu já trabalhei com isso, tenho comprovado, em carteira. Ah, mas precisa. Não discriminava nada disso, era nítido, entendeu? Que, tipo, a pessoa não queria eu, entendeu?”.* (Ingrid) *“Porque eu perdi meu emprego. Tipo assim, né? As coisas começaram a ficar bem trans pra mim. E aí eu perdi meu emprego.”* (Eller, pessoa não-binária/travesti, branca, 27 anos)

## DISCUSSÃO

O presente estudo, primeiro deste tipo realizado na Baixada Santista, descreveu o perfil socioeconômico e demográfico da população trans que residia, trabalhava ou estudava na região. 

O mapeamento da população trans na Baixada Santista utilizou metodologia de recrutamento *online*, que se mostrou de fácil organização e de baixo custo. A resposta ao convite foi positiva, com uma boa adesão de pessoas que completaram o cadastro inicial. Entretanto, em alguns municípios da Baixada Santista, o número de participantes ficou abaixo do esperado. É possível que a distribuição da população trans seja heterogênea nesta região, e fatores como a atuação do movimento social, a presença de rede de serviços de saúde e de assistência social também possam influenciar a participação. 

Uma em cada cinco participantes se identificou como pessoa não-binária, superior ao estudo do Centro de Estudos de Cultura Contemporânea – Cedec (2021), realizado no município de São Paulo em 2021.^6^ Diferenças na autoidentificação em relação ao gênero podem surgir dos desafios de capturar essa variável em pesquisas, dada sua natureza fluida e, em alguns casos, o estágio de transição que as pessoas podem estar vivenciando. Além disso, há questões geracionais e de temporalidade que também podem ter impactado uma proporção de homens trans e pessoas transmasculinas mais próxima à de mulheres trans e travestis.^6^ Outro fator que pode ter contribuído para essa diferença foi a forma alternativa de captação das pessoas, via cadastro *onlin* ^e^.^24^

A proporção de pessoas com idade até 29 anos foi maior na Baixada Santista, cerca de dois terços em comparação à população pesquisada em São Paulo (55%).^6^ O pré-cadastro *online* pode ter proporcionado a maior participação de jovens respondentes observada neste estudo. 

Os processos de autopercepção da identidade trans e início da transição social ocorreram majoritariamente entre 10 e 19 anos e entre 15 e 19 anos, respectivamente. Este resultado corrobora dados do estudo realizado em 2021 no município de São Paulo, que identificou que 70% das participantes já tinham percepção da identidade de gênero entre 5 e 15 anos de idade.^6^

A população que se identificou como preta/parda na região da Baixada Santista foi menor do que no mapeamento realizado pelo Cedec^6^ (42,2% *versus* 57,0%), porém foi maior do que a obtida no estudo TransOdara/São Paulo (24,7%).^25^

Mais de 80% das pessoas participantes haviam concluído o ensino médio, sendo esse valor superior aos resultados observados no mapeamento da população trans do município de São Paulo (63%) e do estudo TransOdara/São Paulo (75%).^6^, A proporção também é mais elevada que entre a população geral com 25 anos ou mais (54,5%).^26^

Cerca de um terço não teve renda no mês anterior, valor este maior do que o do estudo do Cedec (6%). A proporção de pessoas que declararam renda maior do que 3 salários mínimos (10,1%) foi o dobro do verificado no mapeamento realizado pelo Cedec (5,0%)^6^ e no TransOdara/São Paulo (4,3%).^25^

Um décimo das pessoas participantes participantes vivia em situação de moradia instável, percentual inferior ao observado nos demais inquéritos comparados (18% e 13%, respectivamente).^6^, Tais estudos prévios ocorreram durante a pandemia de covid-19, o que pode ter influenciado na diferença dos dados obtidos nessas pesquisas.^6^

Questões relacionadas à formação escolar, trabalho e moradia também estavam presentes na etapa qualitativa. Duas mulheres trans entrevistadas indicaram que saíram ou foram expulsas de casa por conta de questões de gênero, aos 12 e 13 anos de idade, respectivamente. Além disso, houve relatos de dificuldade de permanência na escola e no trabalho por situações de violência verbal e/ou física. A Associação Nacional de Travestis e Transexuais (Antra) estima que 13 anos é a idade média em que mulheres trans e travestis são expulsas de casa pelos pais.^27^ A compreensão e exposição da identidade de gênero é algo que transforma a vivência de pessoas trans, as quais podem começar a sofrer com marginalização social por parte da família,^6^ o que está relacionado à evasão escolar e à dificuldade de acessar o mercado formal de trabalho.^27,9^

Os excertos da etapa qualitativa destacaram que aspectos socioeconômicos – escolaridade, emprego e renda – são permeados centralmente pela identidade de gênero, a partir das percepções das pessoas participantes. Portanto, pesquisas, ações e políticas públicas que visam escolaridade, trabalho e renda na população precisam ser realizadas a partir de variáveis que perpassam a identidade de gênero, como violências no âmbito familiar, escolar e laboral, que impactam o acesso e a permanência no mercado de trabalho, assim como a renda.^22^, Ressalta-se também a importância de melhorar o acesso de pessoas trans, adolescentes e adultas, aos serviços associados às políticas de assistência social, para evitar situações de vulnerabilidade social e violação de direitos,^29^ entendendo-se o impacto da transfobia na relação trabalho-renda.^29^

O Mapeamento da População Trans da Baixada Santista alcançou o objetivo ao qual se propôs de trazer dados sobre as características socioeconômicas e demográficas da população trans da Baixada Santista. 

Neste estudo, empregou-se uma forma de recrutamento predominantemente via internet, por meio do preenchimento de cadastro, cujas vantagens seriam: facilidade de aplicação, disseminação e baixo custo; e a possibilidade de maior alcance, em especial para a população adulta. A desvantagem seria, entre outras, dificultar o alcance de pessoas pouco familiarizadas com a internet e sem acesso a ela. A metodologia empregada neste estudo mostrou-se efetiva para captação da amostra, apesar de que, em alguns municípios, o número de pessoas incluídas ficou abaixo do esperado, embora a equipe de pesquisa tenha realizado a divulgação de forma exaustiva. As limitações do estudo são, junto às desvantagens do recrutamento, uma amostra não probabilística da população trans com adesão espontânea à investigação. Portanto, os resultados obtidos nessa pesquisa podem não ser representativos da população trans como um todo.

Este estudo acessou alguns aspectos socioeconômicos em uma amostra de pessoas trans na Baixada Santista que apresentou como características ser jovem, majoritariamente branca e negra, e mais equilibrada proporcionalmente em relação às identidades de gênero. Verificou-se uma alta frequência de conclusão do ensino médio, porém com mais de 60% das pessoas sem renda ou recebendo menos de 1 salário mínimo. E, em associação com os resultados qualitativos, compreendeu-se que aspectos sociais relacionados à identidade trans permeiam obstáculos na formação escolar e no ambiente laboral.
